# Estimating the Depth of Anesthesia from EEG Signals Based on a Deep Residual Shrinkage Network

**DOI:** 10.3390/s23021008

**Published:** 2023-01-15

**Authors:** Meng Shi, Ziyu Huang, Guowen Xiao, Bowen Xu, Quansheng Ren, Hong Zhao

**Affiliations:** 1 School of Electronics, Peking University, Beijing 100084, China; 2 Department of Anesthesiology, Peking University People’s Hospital, Beijing 100044, China

**Keywords:** deep learning, depth of anesthesia, electroencephalogram, patient state index

## Abstract

The reliable monitoring of the depth of anesthesia (DoA) is essential to control the anesthesia procedure. Electroencephalography (EEG) has been widely used to estimate DoA since EEG could reflect the effect of anesthetic drugs on the central nervous system (CNS). In this study, we propose that a deep learning model consisting mainly of a deep residual shrinkage network (DRSN) and a 1 × 1 convolution network could estimate DoA in terms of patient state index (PSI) values. First, we preprocessed the four raw channels of EEG signals to remove electrical noise and other physiological signals. The proposed model then takes the preprocessed EEG signals as inputs to predict PSI values. Then we extracted 14 features from the preprocessed EEG signals and implemented three conventional feature-based models as comparisons. A dataset of 18 patients was used to evaluate the models’ performances. The results of the five-fold cross-validation show that there is a relatively high similarity between the ground-truth PSI values and the predicted PSI values of our proposed model, which outperforms the conventional models, and further, that the Spearman’s rank correlation coefficient is 0.9344. In addition, an ablation experiment was conducted to demonstrate the effectiveness of the soft-thresholding module for EEG-signal processing, and a cross-subject validation was implemented to illustrate the robustness of the proposed method. In summary, the procedure is not merely feasible for estimating DoA by mimicking PSI values but also inspired us to develop a precise DoA-estimation system with more convincing assessments of anesthetization levels.

## 1. Introduction

Anesthesia is essential to ensuring the successful implementation of surgeries and the safety of patients [1]. The precise control of the anesthesia procedure depends on the reliable monitoring of the depth of anesthesia (DoA), which has been a hot topic for medical researchers in the field [2,3]. An electroencephalogram (EEG) uses scalp electrodes to capture the brain’s electrical activity, which may reflect the effect of anesthetic drugs on the central nervous system (CNS). Therefore, EEGs have been widely used in emotion recognition, depression detection, DoA estimation, etc. [4,5,6,7,8,9,10].

In clinical practice, several EEG-based commercial monitors of DoA have been introduced with the aid of anesthetists. Among these, the bispectral index (BIS) monitor (Aspect Medical Systems, Newton, MA) is the most popular device, representing different anesthetized states using BIS values that range from 0 to 100 [11]. The NEXT Generation SedLine^®^ Brain Function Monitoring (Masimo, Irvine, CA, USA) device has been recently introduced, and its crucial parameter is the patient state index (PSI) [12]. Previous work shows that the agreement between the PSI and BIS is relatively good, and the SedLine monitor is advantageous because it has more channels than the BIS monitor [13].

Over the past years, various EEG-based features have been proposed to assess DoA. Entropy features may be used to measure the complexity and irregularity of signals [14]. Permutation entropy (PE) and sample entropy (SampEn) features are commonly used to estimate DoA [15,16]. Wavelet transform-based features such as wavelet-weighted median frequency and wavelet-coefficient energy entropy have also shown a good performance in DoA estimations [17]. In addition, recent research has revealed a combination of multiple features that could improve DoA estimation. Ortolani et al. [18] combined 13 features to estimate DoA using an artificial neural network (ANN). Shalbaf et al. [19] used an adaptive neuro-fuzzy system with fractal, entropy, and spectral features to assess DoA. Gu et al. [20] extracted 4 features and compared the performances of an ANN and support vector machine (SVM) in a DoA assessment. Their methods successfully distinguished the awake state from other anesthetized states, but the performance results for the deeply anesthetized state were not satisfactory. Moreover, these proposed models are highly dependent on the features of manual design and selection. An EEG is a physiological signal that may be affected by several kinds of noise. Hence, some noise-sensitive features may have a negative effect on the model’s performance or even render it unable to compute.

Deep learning models have been demonstrated to outperform conventional machine-learning models in various fields such as computer vision, natural-language processing, and biomedical-signal processing in their ability to automatically extract high-level features from data [21]. Recently, some researchers have applied deep-learning techniques to estimate DoA. Lee et al. [22] implemented a deep-learning model consisting of long short-term memory (LSTM) and a feed-forward neural network to predict BIS values, surpassing the pharmacokinetic-pharmacodynamic model. Afshar et al. [23] combined deep residual networks (ResNets) and bidirectional LSTM (Bi-LSTM), using EEG signals as the input. Their proposed models surpassed the conventional feature-based models, but the results of the different anesthetized states needed to be more balanced. They did not involve studies that used more channels to predict PSI values. These works should consider the detailed preprocessing of the noise of the raw EEG signal; otherwise, it may lead to poor regression and classification results. A network that is too singular and simple in terms of its structure cannot play the advantages of the end-to-end deep-learning model.

In this paper, we utilized an effective and mature preprocess to remove the noise of the raw EEG signal and propose a deep-learning model to estimate DoA using PSI values as the reference outputs. Our proposed model mainly consists of a revised deep residual shrinkage network (DRSN) and a 1 × 1 convolution network. The DRSN is suitable for dealing with signals disturbed by noise, while the 1 × 1 convolution can increase the representation power and reduce the dimension of neural networks. Our proposed model directly takes EEG signals as inputs and predicts a value ranging from 0 to 100 as a measure of DoA. We extracted 14 features from EEG signals and implement three conventional feature-based models as comparisons. We also compared the performances of our proposed model and the other models in terms of both regression and classification metrics to demonstrate the former’s superiority. Our studies thus offer a new strategy that is both promising and feasible for processing EEG signals for DoA estimation, and they spark inspiration for developing a better DoA-estimation system by integrating PSI or BIS values with expert assessments.

**Figure 1:**
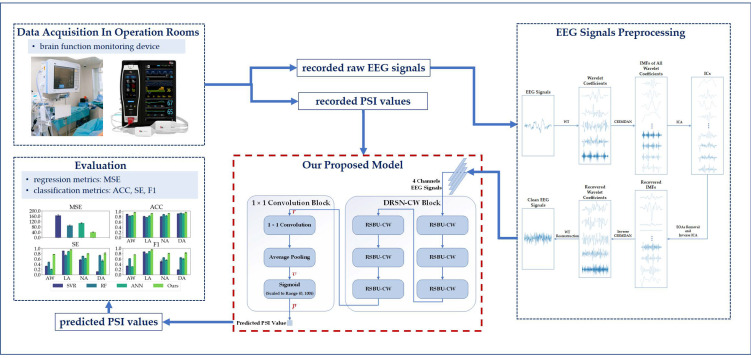
Our workflow.

Our workflow is shown in Figure 1, and the rest of this paper is organized as follows: Section 2 describes the dataset and methodologies. Section 3 presents the experimental settings and results. The discussion is presented in Section 4, and conclusions are given in Section 5.

## 2. Materials and Methods

### 2.1. Dataset

The dataset used in this research, which contains 18 patients aged 66–92 years old, was registered, collected, and provided by Peking University People’s Hospital. During hip fracture repair surgeries, the patients received spinal anesthesia, and midazolam was given to achieve a soothing state. The raw EEG signals were recorded using the NEXT Generation SedLine^®^ Brain Function Monitoring (Masimo, Irvine, CA, USA) device, which was recently introduced into clinical practice and displays PSI as the index of sedation depth. The SedLine EEG sensor consists of 6 electrodes: 1 reference channel (CT), 1 ground channel (CB), and 4 active EEG channels (L1, L2, R1, and R2) placed in the frontal pole. During the midazolam anesthesia, the raw EEG signals are sampled at 178.2 Hz. The dataset we used records 4 channels’ raw EEG signals, PSI values, spectral edge frequency (SEF), burst suppression ratio, electromyographic (EMG) activity, and artifact percentage.

### 2.2. EEG Signals Preprocessing

Raw EEG signals recorded in operation rooms are usually contaminated by electrical noise and other physiological (not brain-related) signals (e.g., eye movements, heartbeats, and muscle activities). Therefore, it is necessary to preprocess the raw EEG signals before the subsequent analysis.

First, we split the raw EEG signals into 4 s long segments with 50% overlap for further processing.

Second, we adopted a bandpass (1–51 Hz) finite impulse response (FIR) filter to remove the electrical noise and baseline drift. Most FIR filters are linear-phase filters and don’t cause a phase distortion or delay distortion of the EEG signals. However, the artifacts, especially electrooculogram artifacts (EOAs) whose magnitude is much higher than that of EEG, often have a spectral overlap with the EEG signals. Hence, it becomes a dilemma where traditional bandpass filters cannot remove EOAs while preserving the desired EEG information.

**Figure 2:**
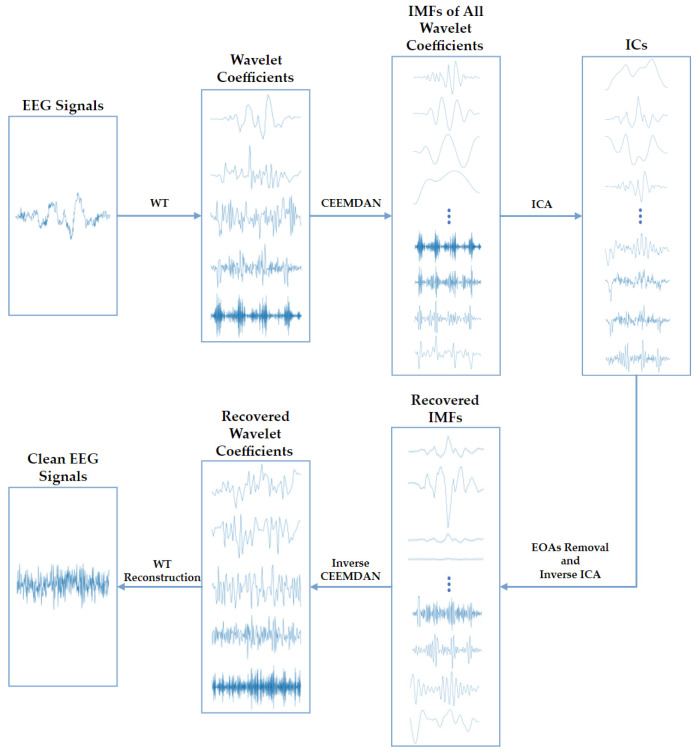
The algorithm flowchart of the WT-CEEMDAN-ICA method used to remove EOAs from EEG signals.

Third, to deal with the dilemma above, we propose an EOA-removing algorithm WT-CEEMDAN-ICA based on wavelet transform (WT), complete ensemble empirical-mode decomposition with adaptive noise (CEEMDAN), and independent component analysis (ICA) technologies [24,25,26]. As depicted in Figure 2, the WT-CEEMDAN-ICA consists of 6 steps: First, the WT technique is used to decompose the raw EEG signals into several wavelet coefficients. The EOAs and EEG components in the wavelet coefficients can be considered a subset of the original EOAs and EEG components. Second, the CEEMDAN technique is adopted to decompose each wavelet coefficient into several intrinsic mode functions (IMFs). Third, the IMFs of each wavelet coefficient are decomposed into multiple independent components (ICs) by using ICA. Fourth, by setting the threshold of the sample entropy of ICs, the ICs of EOAs and EEG components are separated because they are generated by different sources and are independent of each other. Then, the IMFs of the EEG components are recovered by performing the inverse transformation of ICA on the ICs of EEG components. Fifth, the wavelet coefficients are recovered by performing the inverse transformation of CEEMDAN. Finally, the EEG signals without EOAs are reconstructed by performing the inverse transformation of WT.

Finally, we combined the 4 channels of the clean EEG signals of 4 s with the corresponding PSI value as a sample. Therefore, we obtained 22,282 samples in total to evaluate the performance of our models in estimating DoA.

### 2.3. Evaluation Metrics

In this paper, we built several models to estimate DoA in terms of the predicted PSI values and evaluated their performance in terms of both regression and classification metrics. The mean squared error (MSE) was adopted to measure the difference between the predicted and the ground truth PSI values. In addition, we categorized patient states into 4 different anesthetized states according to the corresponding PSI values, including the awake (AW, PSI: 81–100), light anesthesia (LA, PSI: 51–80), normal anesthesia (NA, PSI: 26–50), and deep anesthesia (DA, PSI: 0–25) states. As shown in Table 1, we used the classification accuracy (ACC), sensitivity (SE), and F1-score (F1) to evaluate the models’ performances on different anesthetized states.

In Table 1, N is the number of samples, $\hat{PSI}$ is the predicted PSI value, PSI is the ground truth PSI value, TP is true positive and it equals the number of samples whose actual labels and predicted labels are both positive, TN is true negative and it equals the number of samples whose actual labels and predicted labels are both negative, FP is false positive and it equals the number of samples whose actual labels are negative and predicted labels are positive, and FN is false negative and it equals the number of samples whose actual labels are positive and predicted labels are negative.

### 2.4. Deep Learning Model

#### 2.4.1. Deep Residual Shrinkage Network

**Figure 3:**
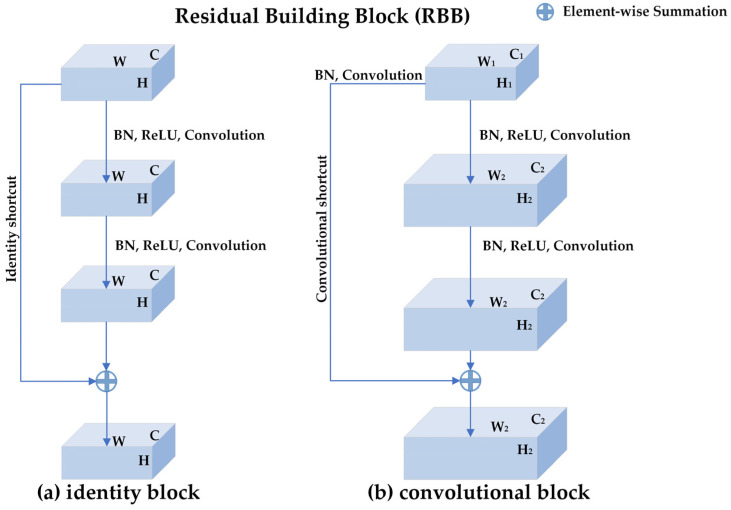
The structure of residual building block (RBB): (**a**) the identity block where the input feature map is the same size as the output feature map. H, W, and C represent the height, width, and channels of the input and output feature map, respectively. (**b**) the convolutional block where the size of the input feature map is different from that of the output feature map. There is a convolution operation and a Batch-normalization operation in the convolutional shortcut for changing the shape of the input. $H_{1}$, $W_{1}$, and $C_{1}$ represent the height, width, and channels of the input feature map, respectively. $H_{2}$, $W_{2}$, and $C_{2}$ represent the height, width, and channels of the output feature map, respectively. An RBB consists of two convolutional layers, two batch normalization (BN) layers, two rectifier linear units (ReLUs) layers, and one shortcut connection.

ResNets are proposed to deal with the degradation problem in deep networks [27]. ResNets introduce the shortcut connections mechanism so that the gradients are not only back-propagated layer by layer but also flow back to the beginning layers directly. As shown in Figure 3, the basic component of ResNets is a residual building block (RBB) which consists of two convolutional layers, two batch normalization (BN) layers, two rectifier linear units (ReLUs) layers, and one shortcut connection. Figure 3a is the identity block where the input feature map is the same size as the output feature map, while Figure 3b is the convolutional block where the size of the input feature map is different from that of the output feature map.

**Figure 4:**
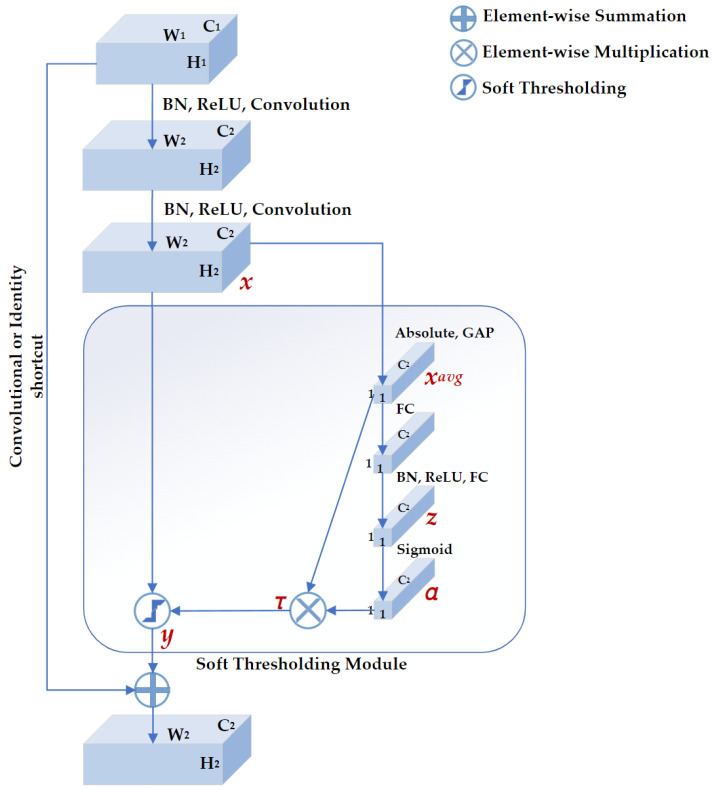
The structure of residual shrinkage building unit with channel-wise thresholds (RSBU-CW). $H_{1}$, $W_{1}$, and $C_{1}$ represent the height, width, and channels of the input feature map, respectively. $H_{2}$, $W_{2}$, and $C_{2}$ represent the height, width, and channels of the output feature map, respectively. There is a soft thresholding module in RSBU-CW. $x_{avg}$, *z*, and *α* are the indicators of the feature maps used to determine the threshold *τ*. *x* and *y* are the input and output feature maps of the soft thresholding module, respectively.

DRSN is a deep learning method that integrates soft thresholding as trainable shrinkage functions inserted into the ResNets. The DRSN forces the unimportant features to be zeros so that the extracted high-level features become more discriminative. Previous experimental results have demonstrated that the DRSN is not only capable of improving the discriminative feature learning ability but is also applicable when dealing with various signals that are disturbed by noise [28]. As depicted in Figure 4, the basic component of DRSN with channel-wise thresholds (DRSN-CW) is a residual shrinkage-building unit with channel-wise thresholds (RSBU-CW). The RSBU-CW is different from the RBB in that the RSBU-CW is distinguished by a special module for estimating thresholds used in soft thresholding. The special module mainly consists of a global-average pooling (GAP) layer, a BN layer, a ReLU layer, a sigmoid layer, and a two-layer fully connected network. The module takes the feature map x as its input to generate a 1D threshold vector τ. The values of τ are positive and kept in a reasonable range so that the RSBU-CW can prevent the output features from being all zero and eliminate noise-related information. The process of the module is expressed as follows:

(1) $$\alpha_{c}=\frac{1}{1+e^{-z_{c}}}$$

(2) $$\tau_{c}=\alpha_{c}\;\cdot x_{avg}$$

(3) $$y_{h,\;w,c}=\left\{\begin{array}{c} x_{h,w,c}-\tau_{c},\;x_{h,w,c}>\tau_{c}\;\\ 0,-\tau_{c}\leq x_{h,\;w,c}\leq \tau_{c}\\ x_{h,w,c}+\tau_{c},x_{h,w,c}<-\tau_{c} \end{array}\right.$$

where h, w, and c are the indexes of height, width, and channel of the input feature map x, and the output feature map y, respectively, $z_{c}$ is the feature at the *c*th neuron of the two-layer fully connected network, $\alpha_{c}$ is the *c*th scaling parameter after the sigmoid layer, and $\tau_{c}$ is the threshold of the *c*th channel.

#### 2.4.2. 1 × 1 Convolution

**Figure 5:**
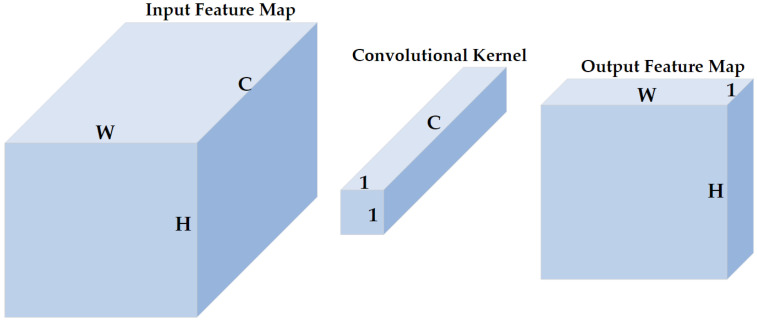
The illustration of 1 × 1 convolution. H, W, and C represent the height, width, and channels of the input feature map, respectively. A 1 × 1 convolution does not change the height or width but the number of channels of inputs.

1 × 1 convolution was proposed to increase the representation power and reduce the dimension of neural networks [29,30]. As shown in Figure 5, the size of the input feature map of the 1 × 1 convolutional layer is H×W×C (H, W, and C represent the height, width, and channels, respectively, and are henceforth represented as such in the rest of this paper), the size of the 1 × 1 convolutional kernel is 1×1×C, and the size of the corresponding output feature map is H×W×1. The 1 × 1 convolution does not change the height or the width of feature maps but reduces the number of channels. Therefore, a 1 × 1 convolution is effective for dimensionality reduction, adding additional non-linearity to networks, and creating smaller convolutional neural networks that retain a higher degree of accuracy.

#### 2.4.3. Proposed Regression Model

**Figure 6:**
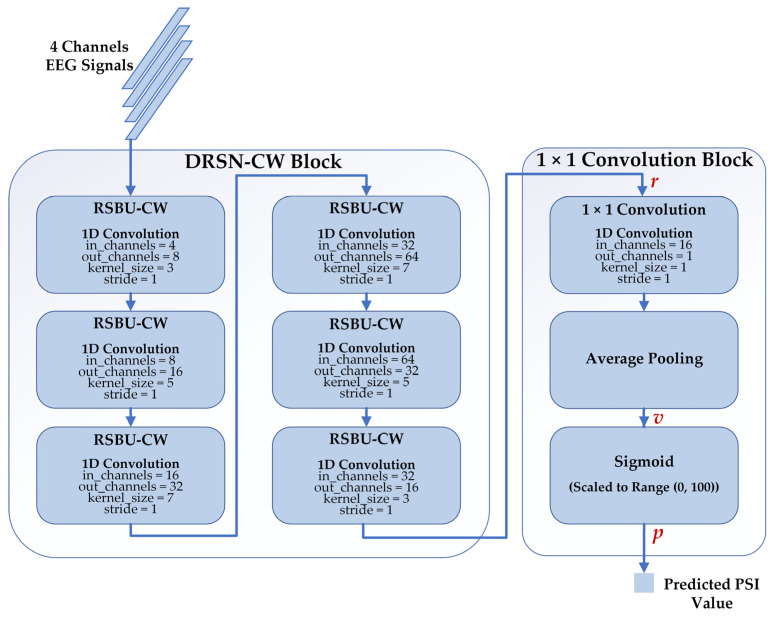
The structure of our proposed model consists of the DRSN-CW block and 1 × 1 convolution block. The inputs of our proposed model are 4 channel-EEG signals, and the outputs are the corresponding predicted PSI values.

We proposed a deep learning regression model to estimate the depth of anesthesia based on DRSN-CW and 1 × 1 convolution. The architecture of our proposed model is depicted in Figure 6. There are two blocks in the model: the DRSN-CW block and the 1 × 1 convolution block. We implemented the RSBU-CW with 1D convolutions because the input EEG signals of each channel are 1D time series. Besides this, we replaced the activation function ReLU in the RSBU-CW with the exponential linear unit (ELU) for better performance. The DRSN-CW block was used to automatically extract high-level feature representations from the EEG signals. In general, fully connected networks are adopted to predict the desired values using the final representations. However, the parameters of a fully connected network are usually more than half of those of the whole model, resulting in the risk of overfitting and expensive computation. Therefore, we used the 1 × 1 convolution instead of the fully connected network to predict the PSI values. The size of the final representation r was 1×W×16, and the 1D convolution layer reduced the dimension of r into 1×W×1. Then, an average pooling layer was used to generate a single value v. Finally, the predicted PSI value p was generated with a Sigmoid function and scaled to the range of (0, 100) as follows:

(4) $$p=\frac{100}{1+e^{-v}},$$

and we used MSE as the loss function of our proposed regression model.

### 2.5. Conventional Models

#### 2.5.1. Features Extraction

The conventional models usually use extracted features instead of the clean EEG signals as inputs. Therefore, we extracted several features relating to PSI from the EEG samples according to what Drover et al. [12] propose.

- Band Power

The recorded EEG signals consist of 4 different channels (FP1, FP2, F7, and F8, according to the international 10–20 system [31]). We computed the power spectral density for each frequency band with the MNE-Python package [32]. The EEG signals can be divided into five frequency bands (δ [1–4 Hz], θ [4–8 Hz], α [8–14 Hz], β [14–31 Hz], and γ [31–51 Hz]) according to their frequency ranges. Then, we computed the band powers of each frequency band using the multitaper spectral-analysis method [33]. Furthermore, the relative band power of a specific-frequency band could be calculated by dividing it by the total band power.

- Spectral Edge Frequency

The spectral edge frequency (SEF) is a popular measure used in EEG monitoring [34]. SEF95 is the frequency below which 95% of the total power of a given signal is located. We computed the SEF95 in the left and right hemispheres, respectively.

- Sample Entropy

Approximate entropy is a measure describing the complexity and regularity of time series, while sample entropy is a similar but more accurate method to approximate entropy [35,36]. Sample entropy has been used to estimate DoA and has achieved good results [16]. Based on the existing research’s parameter settings, we calculated each channel’s sample entropy values as the last 4 features.

In total, we extracted 14 features from the EEG signals, including the total power in the frontopolar region, the total power in the left hemisphere, the total power in the right hemisphere, the band power changes in δ, the band power changes in θ, the band power changes in α, the band power changes in β, the band power changes in γ, the SEF95 in the left hemisphere, the SEF95 in the right hemisphere, and the sample entropy values of 4 channels, respectively.

#### 2.5.2. Conventional Regression Models

This paper used three conventional regression models to estimate DoA as comparisons, including the support vector regression (SVR), random forest (RF), and ANN.

- Support Vector Machine

SVM is a classic supervised machine learning model that analyzes data for both classification and regression tasks [37], and the model for regression tasks is support vector regression (SVR) [38]. Although there are slight differences between SVR and SVM, they share the same core idea of finding a hyperplane that best divides the training samples. In this paper, we used SVR with radial basis function kernel.

- Random Forest

RF is a classic supervised learning algorithm that uses an ensemble learning method for classification and regression tasks. RF operates by constructing multiple decision trees and outputting the mean prediction of the individual trees. In this paper, we used 300 trees to train the model.

- Artificial Neural Network

ANN is a computing system inspired by the biological neural networks that constitute animal brains [39]. ANN is a nonlinear statistical model that learns complex relationships between inputs and outputs to find new patterns. In this paper, we implemented an ANN of the structure 14–64–16–1 to predict the PSI values.

## 3. Results

### 3.1. Experimental Settings

**Figure 7:**
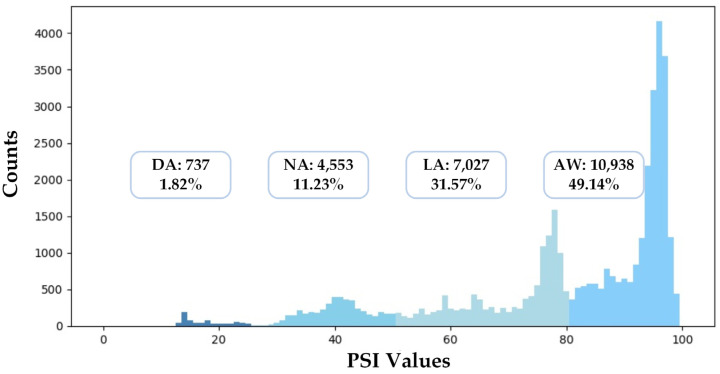
The data distribution of the dataset used in this study.

In this section, we describe the experimental settings in detail. To evaluate the performances of different models, we conducted a five-fold cross-validation: First, we shuffled the whole dataset randomly and split the dataset into five groups. Second, for each group, we took the dataset as the test set, trained models on the other 4 groups, and evaluated models on the test set. Finally, we summarized the results of the five-fold cross-validation with the mean and variance of all metrics. Besides this, we also implemented a cross-subject validation as a supplement. Figure 7 illustrates the distribution of the dataset used in this study. The numbers of samples of different anesthetized states are unbalanced: 49.14% of samples belong to AW, while only 3.31% of samples belong to DA.

Each EEG sample contains 4 channels of 4 s EEG signals whose dimension is 4×712. The amplitudes and variances of EEG signals among different individuals and situations could vary greatly. Therefore, the data standardization was adopted to make our proposed model converge faster and generalize better: First, we transformed the EEG signals of the train and test sets into a 1D vector that has 2848 columns. Second, we computed each column’s mean and standard deviation on the train set. Third, we standardized each column by subtracting its mean and dividing it by its standard deviation on both the train and test sets. Finally, we reshaped the 1D vectors into EEG signals whose dimension is 4×712. A similar data standardization was applied to the samples containing 14 extracted features for the conventional feature-based models.

We implemented our proposed model and the ANN model with PyTorch [40]. The Adam optimization was applied to minimize the MSE loss function. We set the batch size as 64, the initial learning rate as 0.005, and the maximum of epochs as 256. The learning rate decreased by 10% every 20 epochs, and we used L2 normalization to prevent overfitting. We implemented the SVR and RF models with Scikit-learn [41].

### 3.2. Experimental Results

**Figure 8:**
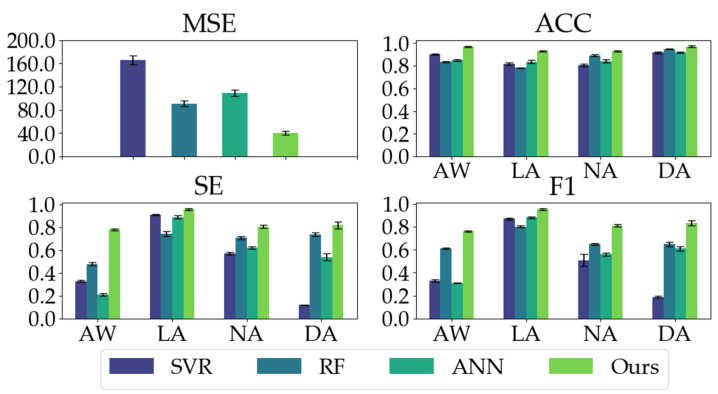
The classification performances (ACC, SE, and F1) of all the models on different anesthetized states (AW, LA, NA, and DA) and the regression performance (MSE) of all the models.

We compared our proposed model with three conventional models. As shown in Table 2, our proposed model has good regression performance and classification ability. In the MSE metric, the mean and STD of our proposed model are significantly less than the conventional models, indicating our proposed model’s impressive regression and generalization performance. Our proposed model yields the highest ACC, SE, and F1 in the classification metrics, especially the ACC and SE values (ACC: 0.9503, SE: 0.8411) in comparison with conventional models (ACC ≤ 0.8640, SE ≤ 0.6685). Moreover, as depicted in Figure 8, our proposed model exhibits the most balanced performance for different anesthetized states.

**Figure 9:**
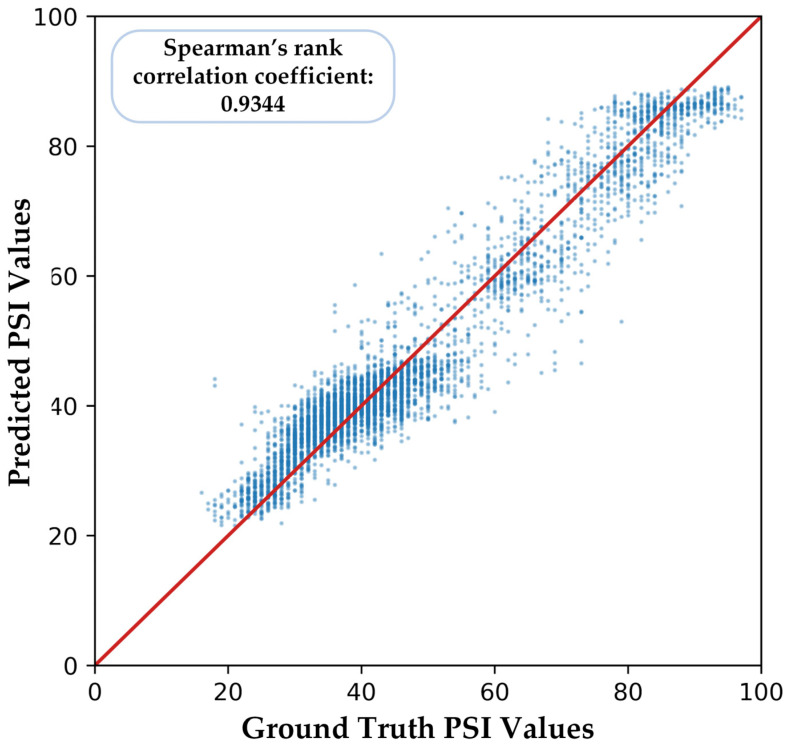
Part of the predicted PSI values of our proposed model. The red line represents the ideal prediction model where the predicted PSI values equal the ground truth PSI values exactly.

We illustrate one of the five-fold cross-validation results in Figure 9. There is a relatively high similarity between the ground truth PSI values and the predicted PSI values of our proposed model, and the Spearman’s rank correlation coefficient is 0.9344.

**Figure 10:**
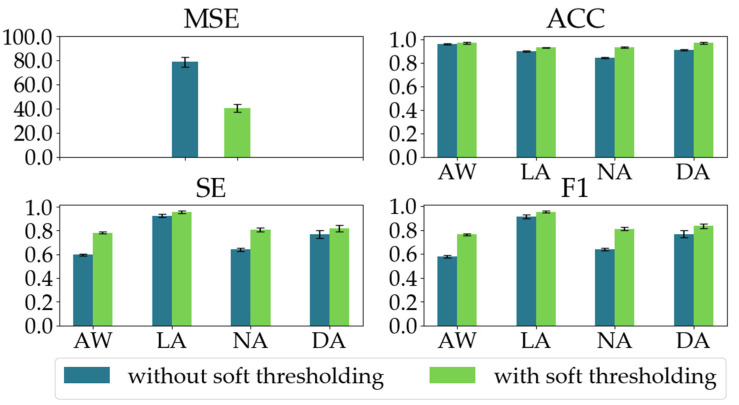
The regression and classification performances of the two models in the ablation experiment on the soft thresholding module in the RSBU-CW.

To demonstrate the effectiveness of the soft thresholding module in the RSBU-CW, we conducted an ablation experiment. We evaluated our proposed model’s regression and classification performances with and without the soft thresholding module in the RSBU-CW. As shown in Figure 10, when the soft thresholding module is ablated, the MSE increases by 38.33, and the classification performances significantly decline as well.

In addition, to further illustrate the effectiveness and robustness of the model we proposed, we conducted a 5-fold cross-validation, which is cross-subject. First, we divided all the subjects into five groups. Specifically, there are four subjects (4122 samples in total) in Group A, four (4271 samples in total) in Group B, four (4590 samples in total) in Group C, three (4793 samples in total) in Group D, and three (5056 samples in total) in Group E.

**Figure 11:**
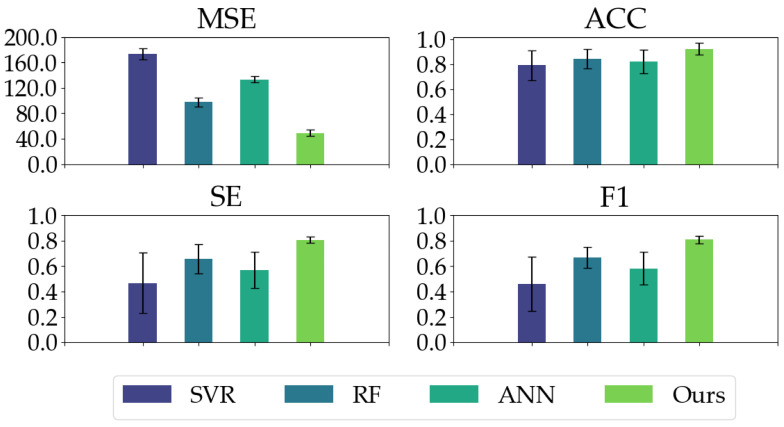
The classification performances (ACC, SE, and F1) of all the models on different anesthetized states (AW, LA, NA, and DA) and the regression performance (MSE) of all the models in cross-subject validation.

Similarly, for each group separately, we took it as the test set, trained models on the other four groups, and evaluated models on the test set. Finally, we summarized the results of the five-fold cross-validation with the mean and variance of all metrics. The results are shown in Table 3 and Figure 11. As can be seen, our proposed model still achieves a better performance in both regression and classification. More precisely, even for the best-performing random forest among the three conventional models, its mean square error is much higher than ours.

**Figure 12:**
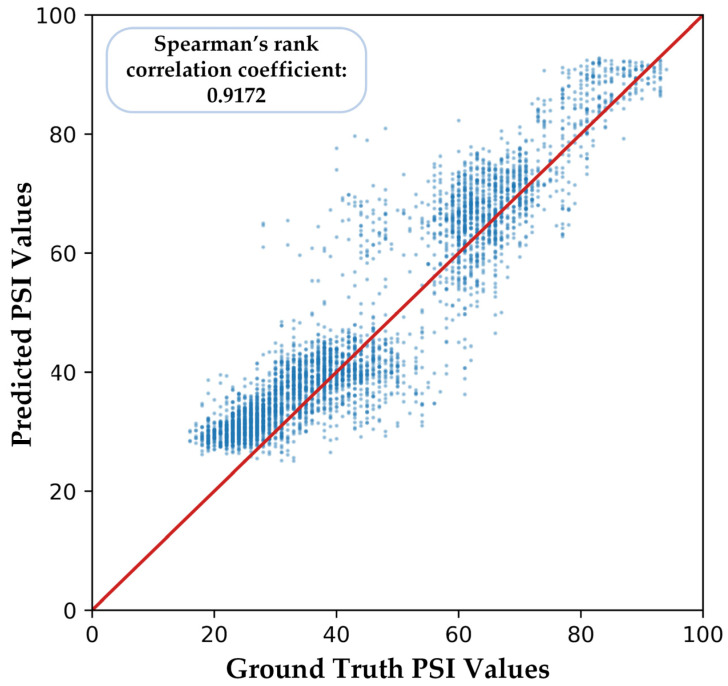
Part of the predicted PSI values of our proposed model in cross-subject validation.

Similarly, we computed Figure 12 to show the correlation between predicted PSI values and ground truth, and Spearman’s rank correlation coefficient is 0.9172, which is still significant.

## 4. Discussion

In previous studies, most researchers used the EEG-based features with conventional models (e.g., ANN, RF, SVR) to estimate DoA in terms of BIS values. However, the regression and classification performances could have been more satisfactory. Besides, the performances of different anesthetized states needed to be more balanced. Thanks to the development of deep learning methods, some researchers adopted deep learning models to process EEG signals directly to assess DoA. The results of deep learning models were better than that of conventional models. These conventional machine-learning methods are based on various features extracted manually [42]. In comparison, the highlight of our proposed model, which mainly consists of the DRSN-CW and the 1 × 1 convolution networks, is that the model can automatically extract features in the training process. Therefore, the trouble and difficulty of manually extracting features are resolved.

The actual experimental results also demonstrate the superiority of our proposed model over other traditional methods. Furthermore, our proposed model achieves the most balanced results for different anesthetized states among all the models, especially for DA, even though there is a dilemma of limited subjects. In addition, we conducted an ablation experiment to demonstrate the effectiveness of the soft thresholding module for EEG-signal processing. Therefore, our proposed model is a promising and feasible method for estimating DoA.

There are several noteworthy points of this research:

1. The recorded raw EEG signals are usually contaminated by electrical noise and other physiological signals. We used bandpass finite filters to remove electrical noise, and the WT-CEEMDAN-ICA algorithm to extract clean EEG signals.
2. We adopted deep learning models to extract discriminative features automatically instead of extracting features manually from EEG signals.
3. To improve our proposed model’s generalization ability and convergence speed, we standardized the EEG signals.
4. DRSN-CW can deal with signals disturbed by noise, which is suitable for EEG-signal processing.

The 1 × 1 convolution network has much fewer parameters than a fully connected network, decreasing the overfitting risk while retaining better performance. Our proposed deep learning model is capable of mimicking PSI values and distinguishing different anesthetized states by directly processing EEG signals, indicating that deep learning methods have tremendous advantage over conventional methods in processing EEG signals to estimate DoA. This research provides inspiration to develop an accurate and reliable DoA assessment system beyond the proprietary PSI or BIS algorithm. Although we used PSI values as DoA labels in this study, our proposed model is not limited to mimicking PSI values. By combining PSI or BIS values with expert assessments of anesthetized levels and building a large-scale DoA dataset, deep learning methods could be evaluated and improved from a more comprehensive perspective. Therefore, directly processing EEG signals with deep learning models is a promising and feasible method to estimate DoA.

## 5. Conclusions

Reliable DoA monitoring is essential for surgeries. For this purpose, we utilized an effective preprocess for noise filtering and propose a deep learning model, mainly consisting of the DRSN-CW and 1 × 1 convolution networks, to estimate DoA in terms of PSI values. We also compared our proposed model with three conventional feature-based models on the dataset of 18 patients. The experimental results show that our proposed model remarkably surpasses conventional models in regression and classification performances. The results of the ablation study and cross-subject validation further illustrate the robustness and structural advantages of the model. Deep learning models are promising and feasible to assess DoA during surgery.

At the same time, we also realize that the information contained in a single kind of physiological signal is limited, which determines the upper limit of the performance of our proposed model. Therefore, to develop a more accurate and reliable DoA assessment system in the future, we will include more signals (such as ECG, EMG, blood pressure, etc.) besides EEG. We will build a larger DoA dataset that combines the PSI values and expert assessments of anesthetized levels as DoA labels, thereby improving our proposed deep learning model.

## Figures and Tables

**Table 1:** Several regression and classification evaluation metrics.

Metric	Formula	Description
MSE (Regression)	$\frac{1}{N}\times \sum_{1}^{N}{\left(\hat{PSI}-PSI\right)}^{2}$	Mean Squared Error
ACC (Classification)	$\frac{TP+TN}{TP+FP+FN+TN}$	Accuracy
SE (Classification)	$\frac{TP}{TP+FN}$	Sensitivity
PR (Not used directly in this paper)	$\frac{TP}{TP+FP}$	Precision
F1 (Classification)	$2\times \frac{SE\times PR}{SE+PR}$	F1-score

**Table 2:** The regression and classification results (mean ± STD) of our proposed model and three conventional models. The mean squared error (MSE) result is the average of the five-fold cross-validation where we split all the samples into five groups, four groups are used as the train set, and one group is used as the test set for each cross-validation. The accuracy (ACC), sensitivity (SE), and F1-score (F1) results are the macro-averaging (we compute the metrics independently for each anesthetized state and then take the average) results of the 4 different anesthetized states.

Metrics	SVR	RF	ANN	Our Proposed Model
MSE	166.02 ± 7.77	90.95 ± 4.88	109.20 ± 5.80	40.35 ± 3.22
ACC	0.8596 ± 0.0574	0.8640 ± 0.0720	0.8606 ± 0.0380	0.9503 ± 0.0224
SE	0.4825 ± 0.3391	0.6685 ± 0.1266	0.5650 ± 0.2801	0.8411 ± 0.0790
F1	0.475 ± 0.2941	0.6770 ± 0.0840	0.5901 ± 0.2337	0.8395 ± 0.0812

**Table 3:** The regression and classification results (mean ± STD) of our proposed model and three conventional models in cross-subject validation.

Metrics	SVR	RF	ANN	Our Proposed Model
MSE	173.22 ± 8.56	97.56 ± 6.88	133.49 ± 5.40	49.22 ± 4.62
ACC	0.7908 ± 0.1187	0.8420 ± 0.0765	0.8216 ± 0947	0.9203 ± 0.0470
SE	0.4675 ± 0.3391	0.6575 ± 0.1266	0.5700 ± 0.1414	0.8054 ± 0.0243
F1	0.4599 ± 0.2132	0.6670 ± 0.0821	0.5852 ± 0.1274	0.8070 ± 0.0306

## Data Availability

The code of our proposed model will be available after acceptance, and the dataset of this study is available from the corresponding author upon reasonable request.

## References

[1] Hajat Z., Ahmad N., Andrzejowski J. (2017). The role and limitations of EEG-based depth of anaesthesia monitoring in theatres and intensive care. Anaesthesia.

[2] Kent C., Domino K.B. (2009). Depth of anesthesia. Curr. Opin. Anaesthesiol..

[3] Fahy B.G., Chau D.F. (2018). The technology of processed electroencephalogram monitoring devices for assessment of depth of anesthesia. Anesth. Analg..

[4] Aydemir E., Tuncer T., Dogan S., Gururajan R., Acharya U.R. (2021). Automated major depressive disorder detection using melamine pattern with EEG signals. Appl. Intell..

[5] Loh H.W., Ooi C.P., Aydemir E., Tuncer T., Dogan S., Acharya U.R. (2022). Decision support system for major depression detection using spectrogram and convolution neural network with EEG signals. Expert Syst..

[6] Tasci G., Loh H.W., Barua P.D., Baygin M., Tasci B., Dogan S., Acharya U.R. (2022). Automated accurate detection of depression using twin Pascal’s triangles lattice pattern with EEG Signals. Knowl.-Based Syst..

[7] Xiao G., Shi M., Ye M., Xu B., Chen Z., Ren Q. (2022). 4D attention-based neural network for EEG emotion recognition. Cogn. Neurodynamics..

[8] Liang Z., Wang Y., Sun X., Li D., Voss L.J., Sleigh J.W., Li X. (2015). EEG entropy measures in anesthesia. Front. Comput. Neurosci..

[9] Saadeh W., Khan F.H., Altaf M.A.B. (2019). Design and implementation of a machine learning based EEG processor for accurate estimation of depth of anesthesia. IEEE Trans. Biomed. Circuits Syst..

[10] Khan F.H., Ashraf U., Altaf M.A.B., Saadeh W. A patient-specific machine learning based EEG processor for accurate estimation of depth of anesthesia. Proceedings of the 2018 IEEE Biomedical Circuits and Systems Conference (BioCAS).

[11] Gonsowski C.T. (2008). Anesthesia Awareness and the Bispectral Index. N. Engl. J. Med..

[12] Drover D., Ortega H.R. (2006). Patient state index. Best Pract. Res. Clin. Anaesthesiol..

[13] Ji S.H., Jang Y.E., Kim E.H., Lee J.H., Kim J.T., Kim H.S. Comparison of Bispectral Index and Patient State Index during Sevoflurane Anesthesia in Children: A Prospective Observational Study. https://www.researchgate.net/publication/343754479_Comparison_of_bispectral_index_and_patient_state_index_during_sevoflurane_anesthesia_in_children_a_prospective_observational_study.

[14] Li P., Karmakar C., Yearwood J., Venkatesh S., Palaniswami M., Liu C. (2018). Detection of epileptic seizure based on entropy analysis of short-term EEG. PLoS ONE.

[15] Olofsen E., Sleigh J.W., Dahan A. (2008). Permutation entropy of the electroencephalogram: A measure of anaesthetic drug effect. BJA Br. J. Anaesth..

[16] Liu Q., Ma L., Fan S.Z., Abbod M.F., Shieh J.S. (2018). Sample entropy analysis for the estimating depth of anaesthesia through human EEG signal at different levels of unconsciousness during surgeries. PeerJ.

[17] Esmaeilpour M., Mohammadi A. (2016). Analyzing the EEG signals in order to estimate the depth of anesthesia using wavelet and fuzzy neural networks. Int. J. Interact. Multimed. Artif. Intell..

[18] Ortolani O., Conti A., Di Filippo A., Adembri C., Moraldi E., Evangelisti A., Roberts S.J. (2002). EEG signal processing in anaesthesia. Use of a neural network technique for monitoring depth of anaesthesia. Br. J. Anaesth..

[19] Shalbaf A., Saffar M., Sleigh J.W., Shalbaf R. (2017). Monitoring the depth of anesthesia using a new adaptive neurofuzzy system. IEEE J. Biomed. Health Inform..

[20] Gu Y., Liang Z., Hagihira S. (2019). Use of Multiple EEG Features and Artificial Neural Network to Monitor the Depth of Anesthesia. Sensors.

[21] Esteva A., Robicquet A., Ramsundar B., Kuleshov V., DePristo M., Chou K., Dean J. (2019). A guide to deep learning in healthcare. Nat. Med..

[22] Lee H.C., Ryu H.G., Chung E.J., Jung C.W. (2018). Prediction of bispectral index during target-controlled infusion of propofol and remifentanil: A deep learning approach. Anesthesiology.

[23] Afshar S., Boostani R. A Two-stage deep learning scheme to estimate depth of anesthesia from EEG signals. Proceedings of the 2020 27th National and 5th International Iranian Conference on Biomedical Engineering (ICBME).

[24] Castellanos N.P., Makarov V.A. (2006). Recovering EEG brain signals: Artifact suppression with wavelet enhanced independent component analysis. J. Neurosci. Methods.

[25] Mammone N., La Foresta F., Morabito F.C. (2012). Automatic artifact rejection from multichannel scalp EEG by Wavelet ICA. IEEE Sens. J..

[26] Torres M.E., Colominas M.A., Schlotthauer G., Flandrin P. A complete ensemble empirical mode decomposition with adaptive noise. Proceedings of the 2011 IEEE International Conference on Acoustics, Speech and Signal Processing (ICASSP).

[27] He K., Zhang X., Ren S., Sun J. Deep residual learning for image recognition. Proceedings of the IEEE Conference on Computer Vision and Pattern Recognition (CVPR).

[28] Zhao M., Zhong S., Fu X., Tang B., Pecht M. (2019). Deep residual shrinkage networks for fault diagnosis. IEEE Trans. Ind. Inform..

[29] Lin M., Chen Q., Yan S. (2013). Network in network. arXiv.

[30] Szegedy C., Liu W., Jia Y., Sermanet P., Reed S., Anguelov D., Rabinovich A. Going deeper with convolutions. Proceedings of the IEEE Conference on Computer Vision and Pattern Recognition.

[31] Seeck M., Koessler L., Bast T., Leijten F., Michel C., Baumgartner C., Beniczky S. (2017). The standardized EEG electrode array of the IFCN. Clin. Neurophysiol..

[32] Alexandre G. (2013). MEG and EEG data analysis with MNE-Python. Front. Neurosci..

[33] Prerau M.J., Brown R.E., Bianchi M.T., Ellenbogen J.M., Purdon P.L. (2017). Sleep neurophysiological dynamics through the lens of multitaper spectral analysis. Physiology.

[34] Obert D.P., Schweizer C., Zinn S., Kratzer S., Hight D., Sleigh J., Kreuzer M. (2021). The influence of age on EEG-based anaesthesia indices. J. Clin. Anesth..

[35] Pincus S.M. (1991). Approximate entropy as a measure of system complexity. Proc. Natl. Acad. Sci. USA.

[36] Richman J.S., Lake D.E., Moorman J.R. (2004). Sample Entropy. Methods in Enzymology.

[37] Vapnik V. (2013). The Nature of Statistical Learning Theory.

[38] Rodriguez-Perez R., Vogt M., Bajorath J. (2017). Support vector machine classification and regression prioritize different structural features for binary compound activity and potency value prediction. ACS omega.

[39] Shahid N., Rappon T., Berta W. (2019). Applications of artificial neural networks in health care organizational decision-making: A scoping review. PloS ONE.

[40] Paszke A., Gross S., Massa F., Lerer A., Bradbury J., Chanan G., Chintala S. (2019). PyTorch: An imperative style, high-performance deep learning library. Adv. Neural Inf. Process Syst..

[41] Pedregosa F., Varoquaux G., Gramfort A., Michel V., Thirion B., Grisel O., Duchesnay É. (2011). Scikit-learn: Machine Learning in Python. J. Mach. Learn. Res..

[42] Acharya U.R., Oh S.L., Hagiwara Y., Tan J.H., Adeli H., Subha D.P. (2018). Automated EEG-based screening of depression using deep convolutional neural network. Comput. Methods Programs Biomed..

